# A study of photothermal laser ablation of various polymers on microsecond time scales

**DOI:** 10.1186/2193-1801-3-489

**Published:** 2014-08-30

**Authors:** Ralf S Kappes, Friedhelm Schönfeld, Chen Li, Ali A Golriz, Matthias Nagel, Thomas Lippert, Hans-Jürgen Butt, Jochen S Gutmann

**Affiliations:** Max Planck Institute for Polymer Research, D-55128 Mainz, Germany; Deutsches Textilforschungszentrum Nord-West gGmbH, D-47798 Krefeld, Germany; Faculty of Engineering, Hochschule RheinMain University of Applied Sciences, D-65428 Rüsselsheim, Germany; Center for Nanointegration Duisburg-Essen (CENIDE), University Duisburg-Essen, D-45141 Essen, Germany; Functional Polymers, EMPA Swiss Federal Lab. for Materials Science and Technology, CH-8600 Dübendorf, Switzerland; Materials Group, Paul Scherrer Institute, CH-5232 Villigen, PSI, Switzerland

**Keywords:** Temperature measurement, Laser heating, Finite element simulation, Polystyrene, Molecular weight, Poly(α-methylstyrene), Polyimide, Triazene polymer, Ablation threshold

## Abstract

To analyze the photothermal ablation of polymers, we designed a temperature measurement setup based on spectral pyrometry. The setup allows to acquire 2D temperature distributions with 1 μm size and 1 μs time resolution and therefore the determination of the center temperature of a laser heating process. Finite element simulations were used to verify and understand the heat conversion and heat flow in the process. With this setup, the photothermal ablation of polystyrene, poly(α-methylstyrene), a polyimide and a triazene polymer was investigated. The thermal stability, the glass transition temperature T_g_ and the viscosity above T_g_ were governing the ablation process. Thermal decomposition for the applied laser pulse of about 10 μs started at temperatures similar to the start of decomposition in thermogravimetry. Furthermore, for polystyrene and poly(α-methylstyrene), both with a T_g_ in the range between room and decomposition temperature, ablation already occurred at temperatures well below the decomposition temperature, only at 30–40 K above T_g_. The mechanism was photomechanical, i.e. a stress due to the thermal expansion of the polymer was responsible for ablation. Low molecular weight polymers showed differences in photomechanical ablation, corresponding to their lower T_g_ and lower viscosity above the glass transition. However, the difference in ablated volume was only significant at higher temperatures in the temperature regime for thermal decomposition at quasi-equilibrium time scales.

## Introduction

Lasers interacting with polymers are ubiquitous in scientific and industry applications. Several reviews and studies on simulations and experiments have already regarded laser heating effects and thermal laser ablation (Arnold and Bityurin 1999; Zhigilei et al. 2003; Lippert 2004; Brygo et al. 2006). These studies show that the impact of the process temperature on polymer laser ablation in particular for the reduced reaction times close to thermal confinement induced by laser pulses of 10 μs or less may not be underestimated. For shorter pulse duration, in the regime of thermal confinement, drastic superheating of the polymer prior to ablation was suggested in the literature (Sandy Lee et al. 1992; Küper et al. 1993; Hare et al. 1995; Johnson et al. 2009). The temperature was proposed to extensively exceed the phase transition or degradation temperature before material is being ablated. While being superheated, the polymer is supposed to remain metastable. However, it remains unclear how the polymer reacts in the intermediate regime in which thermal energy transfer may not be neglected, but an accumulation of thermal energy may already occur.

A better understanding of the thermal behavior of polymers in this regime would be beneficial. One important industrial example is the computer-to-plate process for printing plates. In this step of the offset printing process, the computer generated information are transferred to a physical image on the printing plate by microsecond laser pulses. The functional coating for such an application mainly consists of polymers. Faster processing speeds and thus shorter illumination times are required in order to speed up the overall process in particular in newspaper printing. Related with faster processing speeds are shorter illumination and thus reaction times.

Polymer properties are typically measured at quasi-equilibrium conditions, allowing time for polymer diffusion and thermal relaxation. Two well-applied examples are thermogravimetry and differential scanning calorimetry. A correlation of the quasi-equilibrium behavior with the behavior under pulsed laser heating is not trivial, but would be very helpful. Characterization on the time scale of laser pulse experiments requires a specially designed setup and sophisticated data analysis. Comparisons at quasi-equilibrium time scales are in contrast standard measurement procedures and can be used to test a great number of polymers or mixtures and to tailor their properties accordingly.

Furthermore, there are several studies in literature addressing the impact of the molecular weight on polymer behavior when pulsed laser heated (Mito and Masuhara 2002; Rebollar et al. 2006; Rebollar et al. 2008). While a general correlation is reported, the underlying mechanism for a prediction of the behavior is not discussed.

The temperature is one of the most important parameters in order to understand and describe photothermal processes. Several methods to measure temperatures were already applied in order to probe the temperature of laser heating processes on polymers. Prominent examples are pump probe experiments on the principle of coherent anti-Stokes Raman scattering (Hare et al. 1995) or on the principle of a molecular thermometer dye, providing a temperature dependent absorption transition at a certain wavelength (Sandy Lee et al. 1992; Wen et al. 1992; Chen et al. 1992; Wen et al. 1993). Both methods rely, however, on the extrapolation of slow time scale temperature behavior, exceeding the calibration temperature range with the laser pulse experiments significantly. Furthermore, laser induced fluorescence was used to probe the temperature by including a dopant in the polymer sample which underwent a thermal reaction (Bounos et al. 2005). The time dependent formation of the fluorescent active product was tracked and the temperature was obtained from a calculation based on the kinetics of the thermal reaction. However, the application of these methods is limited as a number of side reactions of the dopant with functional groups of the polymer disturb the thermal reaction and thus the temperature measurement.

Spectral pyrometry is a temperature measurement method which is applicable for a wide range of sample systems (Magunov 2009), as well as for laser heating processes on polymers, as we have already reported (Kappes et al. 2010; Kappes et al. 2011). The measurement is based on the analysis of the thermal radiation in the range of visible light, theoretically described by Planck’s law. The method does not depend on the nature of the polymer and can therefore be applied to compare various polymers.

In this paper we describe a method to measure the temperature, based on spectral pyrometry, and its application on the laser heating and ablation process for several polymers close to the regime of thermal confinement. The selection of polymers comprises polystyrene, the thermally less stable poly(α-methylstyrene), a thermally more stable polyimide and a triazene polymer. The latter is frequently applied in the laser induced forward transfer (LIFT) or laser direct writing process (Arnold et al. 2011), and was already studied regarding the thermal influence on photochemical ablation based on thermal modeling to deduce the temperature for UV irradiation (Fardel et al. 2008).

The heating and the ablation behavior are discussed and compared to finite element simulation results excluding ablation. Furthermore, the influence of molecular weight is addressed by the application of polystyrene of two different molecular weights. A comparison to quasi-equilibrium experiments, thermogravimetry and differential scanning calorimetry, is conducted in order to correlate the behavior when pulsed laser heated to the quasi-equilibrium time scale behavior.

## Materials and methods

### Experimental details

**Figure 1:**
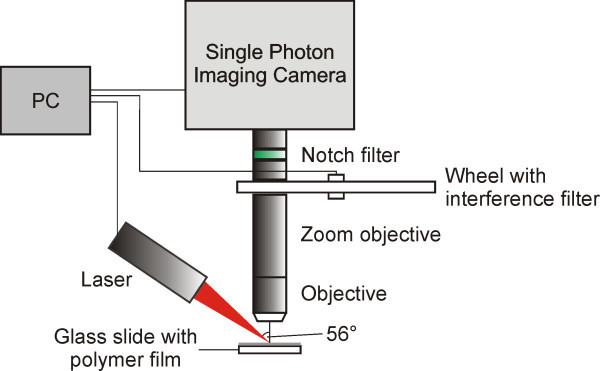
**Scheme of the experimental setup.**

The experimental setup (Figure 1) was described previously (Kappes et al. 2010; Kappes et al. 2011). Briefly, it consists of a 810 nm diode laser, a 100× objective, a zoom objective, a wheel with 12 interference filters and a “Single Photon Imaging Camera” (Theta System Elektronik, GmbH). A time resolution of the camera and therefore of the temperature measurement of 1 μs was achieved. The laser was focused on the surface with a beam diameter of 6.9 × 12.3 μm^2^ ((1/e^2^), measured by Beam Scan model 1180 optical profiler, Photon Inc.). Pulses with the duration of 15 μs were generated via electrical modulation. The pulses were not rectangular on the time scale of 1 μs, but showed a sharks fin characteristic (compare figure five). For the temperature measurements the term “delay time” was used, defined as the time between start of the laser pulse and start of camera integration.

Pulse energies of 0.019 to 1.04 μJ were applied (measured with a laser power sensor (OP-2 Vis, Coherent GmbH)). These pulse energies equal an average fluence (given as experimental parameter in the figures) of 0.028 to 1.56 J/cm^2^ over the 1/e^2^ beam footprint.

The thermal emission data was analyzed on the base of Planck’s law as described previously (Kappes et al. 2010; Kappes et al. 2011). In order to compare several samples, a center temperature based on the average over an area of 1.3 × 1.3 μm^2^, is given instead of the 2D temperature graphs. The error of the center temperature was calculated as reported previously (Kappes et al. 2011). For experimental adjustments and interpretation of the results, especially for deviations from the predicted behavior via simulations, the 2D resolution was however crucial and should not be underestimated in relevance as was shown previously (Kappes et al. 2010; Kappes et al. 2011).

**Figure 2:**
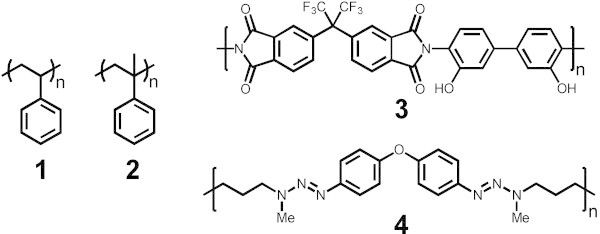
**Chemical structure of different polymers.** Polystyrene **(1)**, poly(α-methylstyrene) **(2)**, polyimide **(3)** and triazene polymer **(4)**.

**Figure 3:**
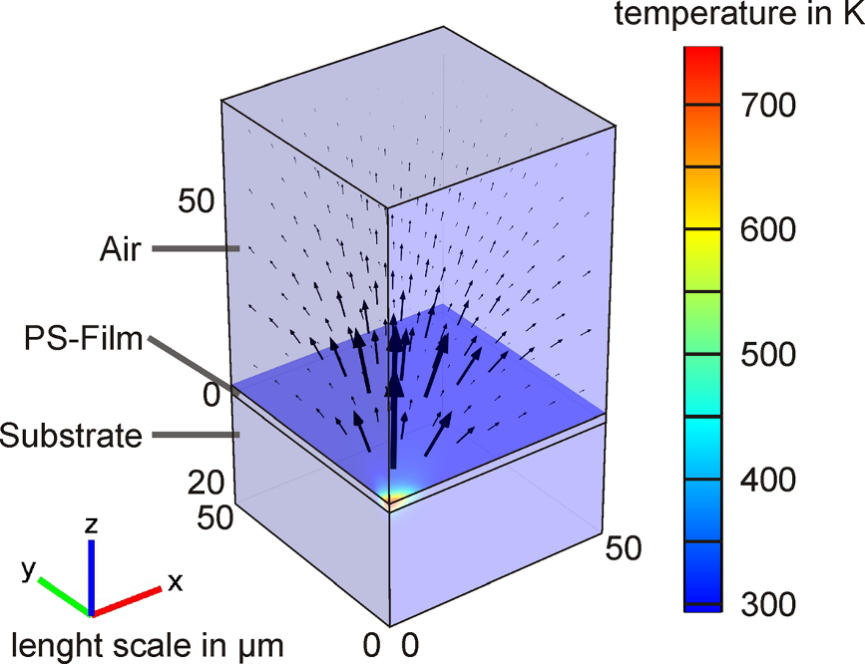
**Scheme of the simulation cell.**

**Figure 4:**
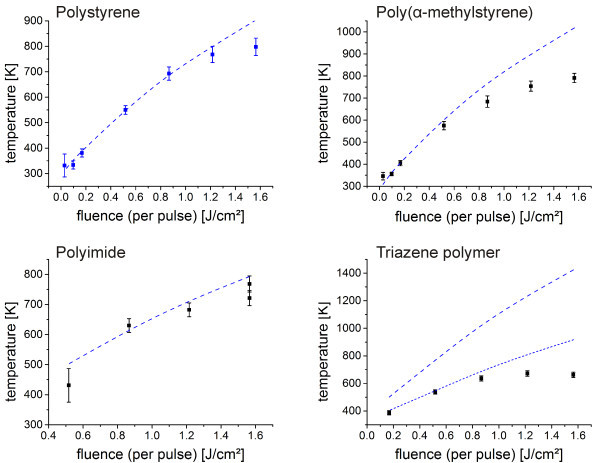
**Center temperature vs. fluence for various polymers (squares) including finite element simulation results for a polystyrene film with according absorbance and film thickness (blue dashed line).** The given temperature corresponds to a delay time of 14 μs with a 15 μs laser pulse and an integration time of 1 μs. The simulations were conducted neglecting ablation. Only for the triazene polymer a simulation with a constant loss factor of 50% was added (blue short-dashed line). The properties of the polymer films are given in table two.

Polystyrene of three different molecular weights (M_n_ = 3,900 g/mol, 158,000 g/mol and 314,000 g/mol, with PDI = 1.12, 1.06 and 1.07, measured by GPC, PS standard) and poly(α-methylstyrene) (M_n_ = 76,500 g/mol, PDI 1.03, measured by GPC, PS standard) were prepared by anionic polymerization. The polyimide (Figure 2, (3), M_n_ = 746 g/mol, M_w_ = 2,350 g/mol, PDI = 3.15, measured by GPC, PS standard) was prepared by condensation polymerization of 2,2′-bis-(3,4-dicarboxyphenyl) hexafluoropropane dianhydride and 3,3′-hydroxy-4,4′-diaminobiphenyl according to the procedure of Hahm et al. (Hahm et al. 2009). The triazene polymer, prepared by interfacial polycondensation from the bis-diazonium salt of bis(4-aminophenyl) ether and 1,6-bis(methylamino)-hexane, (Figure 2, (4)) was synthesized according to a procedure described previously (Stebani et al. 1993; Nagel et al. 2007).

All polymers were transparent at the applied laser wavelength. Therefore the organic NIR-dye N,N’-bis(2,6-diisopropylphenyl)-1,6,11,16-tetrakis(p-tert-octylphenoxy)-8(9),18-dibromoquaterrylene-3,4:13,14-tetracarboxylic acid diimide was added to the polymer solutions prior to the coating procedure to ensure sufficient absorption at the laser wavelength and thus sufficient energy input.

Thin polymer films of polystyrene and poly(α-methylstyrene) were prepared via blade coating from tetrahydrofuran solutions. Concentrations of 50–100 mg/ml of the polymers were used in order to produce films of 1–3 μm thickness. The dye concentration was adjusted accordingly to achieve a 4% ratio in respect to the polymer, polystyrene respectively poly(α-methylstyrene). Polyimide films were produced via spin coating from cyclopentanone solutions. A concentration of 4.2 mg/ml of the polymer (saturation limit) was applied and a high amount of dye was added (36% in respect to the polymer) to ensure sufficient absorption at the laser wavelength for the smaller thicknesses of the polyimide films. The triazene polymer was spin coated from a solvent mixture of chlorobenzene and cyclohexanone. A dye polymer ratio of 10% was used. In this case, thicker films than for the polyimide could be prepared. However, in comparison to the polystyrene and the poly(α-methylstyrene), the films are significantly thinner. Thicker spin coated films of about 1 μm typically suffer in quality. Therefore a lower thickness was chosen and the dye polymer ratio was increased in order to maintain sufficient laser light absorption. All polymer films were prepared on standard glass slides.

The polymer surfaces were examined after illumination by confocal white light profilometry (μsurf, Nanofocus AG) equipped with an UMPLFL 100× objective. Thermogravimetric (TGA 851, Mettler Toledo GmbH) and scanning calorimetry analysis (DSC 30 and DSC 822, Mettler Toledo GmbH) of the various polymers were conducted with a heating rate of 10 K/min. Absorption measurements of the polymer films were performed with a standard UV–vis spectrometer (spectrometer Lambda 900, Perkin Elmer).

### Simulation details

Finite element simulations were carried out with the simulation software Comsol Multiphysics version 4.2a (http://www.comsol.de) as reported previously (Kappes et al. 2011). The model geometry consists of a part of the substrate, the polymer layer and an airbox on top of the polymer (Figure 3). The boundary axes were aligned with the half axes of the elliptical laser pulse in order to exploit the symmetry of the problem. Thermal conduction was enabled in every part of the simulation cell. Convection was included in the airbox. Radiation was neglected due to its minor contribution to the heat flux for temperatures below 1000 K.

Only simulations based on polystyrene were conducted. Temperature dependent data for the thermal parameters, such as the thermal conductivity, heat capacity and density, of polystyrene and the substrate was applied as reported in our previous study (Kappes et al. 2011). This data, especially the temperature dependent behavior was lacking for the other polymers. However, the influence of experimental parameters like the film thickness and energy absorption of the polymer-dye films can be understood using the polystyrene based simulations.

The laser pulse was coupled into the polymer film through a volumetric heat source. In the sample surface plane (xy-plane) the intensity distribution was given by a Gaussian profile according to the beam dimensions of 6.9 × 12.3 μm^2^ (1/e^2^). An absorbance according to Lambert-Beer’s law was applied in z direction based on the absorption of the polymer film at the laser wavelength. The time dependence was set according to an interpolated pulse shape. The data for the interpolation was obtained with the setup described above without any filters in the beam path, i.e. via the intensity of the laser stray light on a non-coated substrate. A reflection loss at the air-polymer interface was accounted by calculation of the Fresnel coefficients and subtracted from the input energy. Further reflective phenomena were neglected. Other energy loss mechanisms including for example fluorescence or phosphorescence were also neglected, in agreement with our previous studies (Kappes et al. 2011). For the triazene polymer, a constant loss factor was included additionally in the volumetric heat source in order to allow a better comparison (see Results and discussion - Laser-polymer interaction).

A prismatic mesh was defined by extrusion of a triangular mesh in z-direction. A fine mesh with cell dimensions in the range of 10 nm was defined in the heating center while coarser mesh cells with dimensions of about 1 μm were placed at the outer boundaries. By means of mesh sensitivity tests, it was assured that the maximum discretization error in the computed temperatures were well below 1 K.

## Results and discussion

### Comparison of different polymers

The polymers applied in this study differed in glass transition behavior and in thermal stability (Table 1). Polystyrene, poly(α-methylstyrene) and the triazene polymer showed thermoplastic behavior. The glass transition temperature increased from the triazene polymer (336 K) over polystyrene (371 K) to the poly(α-methylstyrene) (410 K). The polyimide in contrast was a thermoset which showed no glass transition in the range between the room and the degradation temperature.

**Table 1:** **Glass transition temperature (T**_**g**_ **, DSC, midpoint DIN) and the results of a thermogravimetric analysis of the different polymers**

Polymer	T _g_[K]	T _10% loss_[K] ^***a***^	T _20% loss_[K] ^***b***^
Polystyrene (314,000 g/mol)	371	595	611
Poly(α-methylstyrene)	410	545	554
Polyimide	-	572	657
Triazene polymer	336^*c*^	530^*c*^	544^*c*^

^*a*^T_10% loss_ for 10% mass loss, ^*b*^T_20% loss_ for 20%, ^*c*^according to (Nuyken et al. 1995).

In terms of the thermal stability the triazene polymer decomposed at lowest temperatures, followed by the poly(α-methylstyrene) as indicated by the temperatures for 10 and 20% mass loss obtained from a thermogravimetric analysis with low heating rates (10 K/min) (Table 1). Polystyrene showed a higher thermal stability. It started decomposing in thermogravimetry at temperatures 50 K above the temperatures obtained for the triazene polymer and poly(α-methylstyrene). The polyimide showed the highest thermal stability. The initial small mass loss for the polyimide (Table 1, T_10%loss_) can be assigned to the low molecular weight and especially the broad distribution of the molecular weight. A significant amount of the monomer and low molecular weight fractions were present beside the polymer which typically decompose first, resulting in the mass loss at low temperatures.

### Laser-polymer interaction

In order to investigate the heating and ablation behavior of the polymers, the temperature was measured in dependency of the fluence. Figure 4 shows the temperatures at a delay time of 14 μs, which is typically close to but not necessarily the maximum temperature reached during the laser pulse. Especially for the thicker polymer films with 1–3 μm, the center temperature can easily go 10–30 K higher. The measured center temperature is not solely representing the polymer surface temperature, but the temperature from every heated matter in close proximity to the polymer surface. For higher temperatures at which polymer decomposition takes place, a combination of decomposing polymer, oligomers, monomer, decay products from the monomer decomposition and heated air contributes to the measured temperature.

Additionally the results of the finite element simulations for polystyrene films with identical film thicknesses and absorption were included in the graphs. These simulations include the heat conversion and diffusion but neglect the ablation. In this way, the heating behavior of the polymers can easily be compared to the polystyrene behavior and the influence of ablation can be visualized.

**Figure 5:**
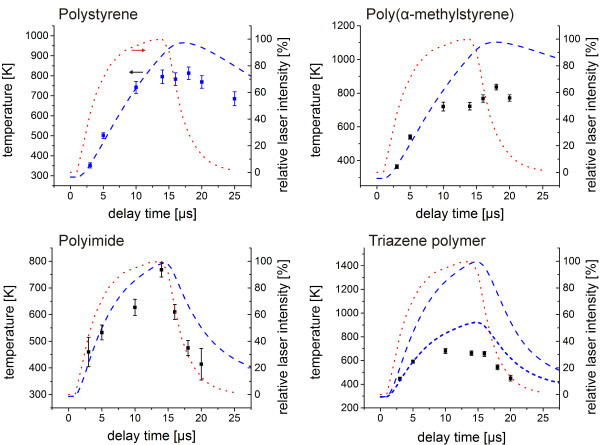
**Center temperature vs. delay time for various polymers (squares) including finite element simulation results (blue dashed line) at a fluence of 1.56 J/cm** ^**2**^ **for a polystyrene film with according absorbance and film thickness.** The simulations were conducted assuming no energy loss. Only for the triazene polymer a simulation with a constant loss factor of 50% was added (blue short-dashed line). Additionally the relative laser intensity profile is included in the graph (red dotted line, right axis).

An increase in temperature with increasing fluence was found, experimentally and by simulations. For higher fluences smaller increases in temperature were evident until a saturation level was reached. Only the fluence and temperature of the saturation level varied for the different polymer films. The simulations disagree in this point with the experimental data. This can be attributed to polymer ablation, which was neglected in the simulations. In an ablation process the hot material is ejected from the polymer surface. This ejection leads to a significant cooling and thus a depression of the center temperature. Additionally, for most polymers, especially the polyimides or polyolefinic polymer as for example polystyrene and poly(α-methylstyrene) the decomposition is known to be endothermic. Thus, a further temperature depression might be related to the decomposition reaction. The depression was, however, even more pronounced for the triazene polymer, which was reported to decompose exothermic (Nuyken et al. 1995). Therefore for the triazene polymer the effect of ejection of hot material seems to be dominating in the depression of the center temperature.The discrepancy varied between experimental and simulation data for the different polymers. This indicates the differences in ablation behavior. In the case of the triazene polymer a constant loss factor of 50% needed to be introduced to maintain quantitative agreement for low fluences (blue short-dashed line in Figure 4). The use of the polystyrene simulation parameters seems to be an oversimplification in this case. The thermal conductivity and the heat capacity of the triazene polymer as well as their temperature dependence seem to deviate considerably from the values of polystyrene. In contrast, poly(α-methylstyrene) and the polyimide showed a negligible discrepancy and a transfer of the polystyrene parameters could be carried out.In this respect it is not only of interest to compare the temperature at a constant delay time but also to vary the delay time for a constant fluence. The graphs in Figure 5 show the time dependence of the temperature for the highest applied fluence from Figure 4 including the laser intensity profile and the related simulations. In this way, the influence of the input energy respectively the influence of energy conversion and heat transfer on the measured temperatures could be followed. This is an important issue close to the regime of thermal confinement. The thicker polymer films, the polystyrene and the poly(α-methylstyrene) film, showed for example a different time-temperature profile in the simulation compared to the laser intensity profile, while for the thinner polyimide and triazene polymer film the simulated curve closely follows the intensity profile. This can be attributed to the insulation behavior of the polymer films, an effect which gets more pronounced the thicker the film.

Good agreement between experimental and simulation data was found at the start of the laser pulse until a delay time of 5–10 μs depending on the polymer. For higher delay times, the experimentally determined temperatures were below the predicted values of the simulation.The results support the explanation based on the influence of ablation on the depression of the measured temperatures at high fluences in Figure 4. The ablation did not start at the beginning of the pulse. First, a threshold temperature had to be reached. For this heating phase, the simulations agree with the measured temperatures. At a later stage of the pulse, when the threshold temperature was exceeded, ablation occurred, and the increase in temperature was slowed down drastically. Polymers with higher thermal stability, especially the polyimide, showed a less pronounced deviation between measurement and simulation than the polymer types with lower thermal stability. However, all polymers showed a clear deviation at higher delay times indicating that significant ablation took place in all cases.

It has to be stressed, that the temperature dependence of the input parameters for the other polymer types was unknown. Thus all simulations were conducted using the thermodynamic material properties of polystyrene. It was therefore astonishing, that as long as ablation was limited only minor deviations between the simulation and the experimental data were observed. On the other hand, polymers in general, and the applied polymers in particular, do not show major differences in terms of their thermal conductivity, thermal capacity and density. Consequently, it can be concluded that the use of the polystyrene parameters was legitimate. The only exception is the triazene polymer. An additional constant loss parameter needed to be introduced in order to get quantitative agreement until the effect of ablation takes over, which lowers the temperature considerably.

### Surface morphology after laser illumination

**Figure 6:**
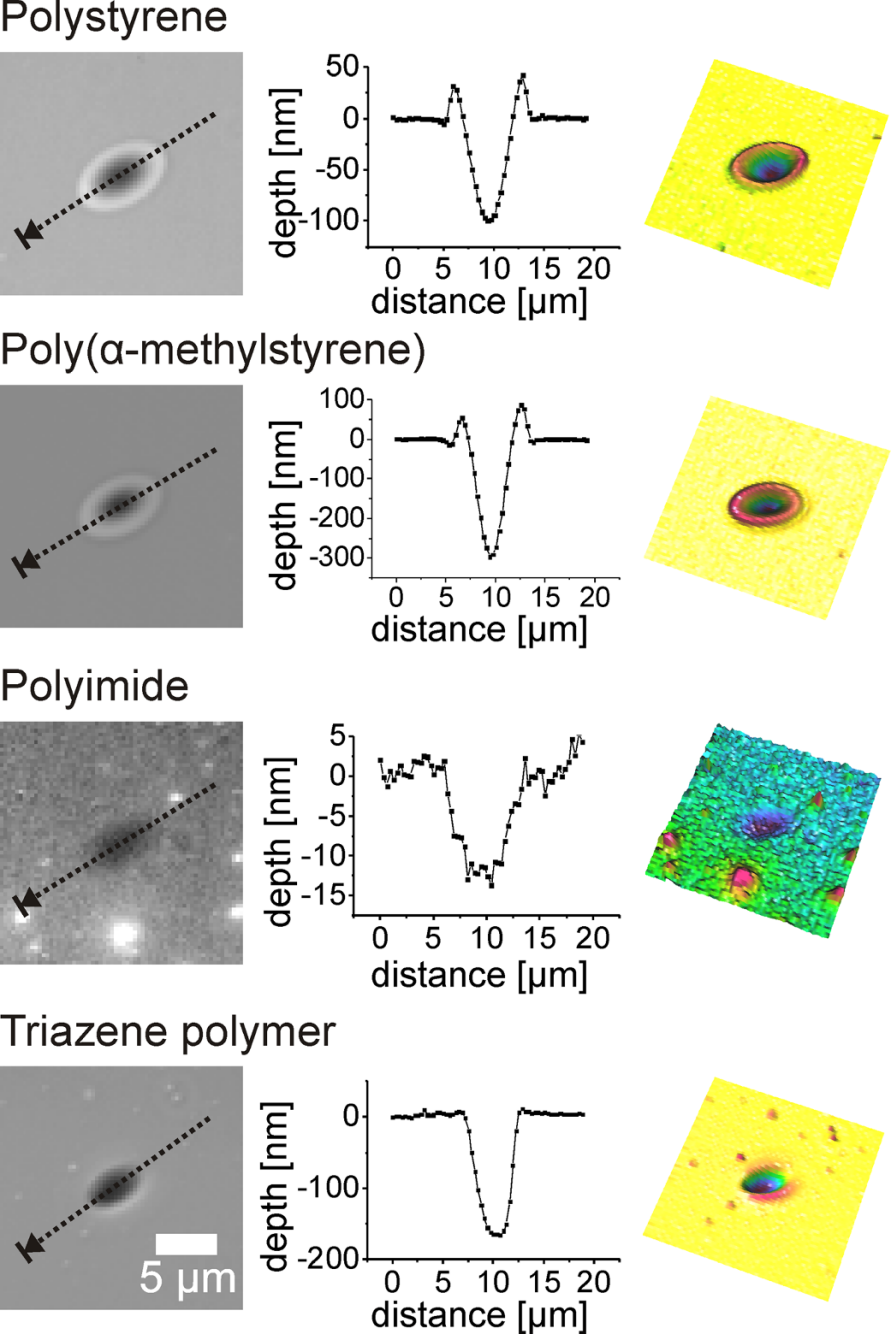
**Surface profiles after laser illumination of various polymers.** The profiles correspond to a fluence of 0.52 J/cm^2^ for the polystyrene (thickness 1930 nm) and the poly(α-methylstyrene) (thickness 3280 nm), a fluence of 1.56 J/cm^2^ for the polyimide (thickness 79 nm) and 1.22 J/cm^2^ for the triazene polymer (thickness 369 nm).

Crater formation was observed upon laser illumination on all polymer films. Figure 6 shows typical crater morphologies for each polymer at fluences well above the ablation threshold. Qualitatively, the appearance of the craters formed on polystyrene and poly(α-methylstyrene) did not differ. Both showed a surrounding rim. Also the crater shape was similar, resembling a Gaussian shape, corresponding to the Gaussian laser footprint. Deeper craters were observed for the poly(α-methylstyrene) compared to the polystyrene. The difference can be attributed to the higher film thickness and absorption of the poly(α-methylstyrene) film (Table 2), but also to the lower thermal stability of the poly(α-methylstyrene) as will be discussed later.

**Table 2:** **Film thickness, dye concentration in respect to the polymer and result of UV–vis absorption measurement at the laser wavelength for the different polymer films**

Polymer	Film thickness [nm]	Dye conc. [%]	Transmission [%]	Absorbance per film thickness [1/m]*10 ^4^
Polystyrene (314,000 g/mol)	1930	4	83.2	4.64
Poly(α-methylstyrene)	3280	4	67.6	5.78
Polyimide	79	36	82.7	104
Triazene polymer	369	10	70.1	41.8

The craters in the polyimide film were only 17 ± 6 nm in depth. This can be attributed to the much lower film thickness, which allowed a more efficient energy transfer from the heated polymer to the substrate. The thermal conductivity of the polymer is about one order of magnitude smaller as compared to the glass substrate. A thick polymer film therefore insulates its own heat dissipation, while the opposite is true in this case. Furthermore, the shape resembled a Gaussian form without a surrounding rim. The noisiness of the line profile was due to the low crater depth, being already close to the detection limit of the applied white light confocal microscopy.

The triazene polymer craters showed a surrounding rim, which was less pronounced compared to the craters in the polystyrene or the poly(α-methylstyrene) films. Furthermore the craters were wider and showed steeper walls. We attribute this change in the crater profile to the fact that for the triazene polymer, the crater depth reached already about half of the total film thickness. In this case, two effects became significant. First, once the film was partly ablated especially in the center of the spot the absorption of the laser energy was reduced. Second, the thinner the remaining polymer film, the more efficient it was cooled by the underlying substrate. This was observed for all polymers when ablation crater depths were in the order of the film thickness. For lower fluences, the Gaussian shape was maintained, for higher fluences, the profile was transformed even further to a flat-top profile.

### Behavior at the ablation threshold

**Figure 7:**
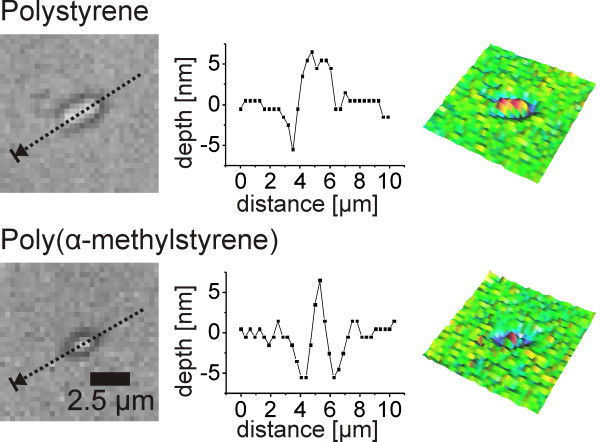
**Surface profiles after laser illumination of polystyrene and poly(α-methylstyrene) below ablation threshold.** The profiles correspond to a fluence of 0.17 J/cm^2^.

Besides the typical ablation craters, a surface deformation of the polymer films was observed on polystyrene and poly(α-methylstyrene) films below the ablation threshold fluence (Figure 7). Protrusions of several nanometers in height were formed. This effect was not observed on the triazene polymer and the polyimide films prior to ablation. The maximum center temperatures for the surface profiles in Figure 7 were 402 ± 16 K for polystyrene and 435 ± 12 K for poly(α-methylstyrene). This corresponds to a temperature, which is 20–30 K above the glass transition temperature of the individual polymer.

For higher fluences and temperatures, photomechanical ablation as reported previously (Kappes et al. 2011) was found for both polymers. Surrounded by non-heated matter the polymers being in the glassy state could not expand when heated up locally. Instead a stress was building up. For temperatures above the glass transition, the volume expands faster (Greiner and Schwarzl 1984). At the same time the viscosity is reduced significantly. This leads to a surface deformation if the stress is limited or to ablation if a threshold stress level is exceeded. In principle this mechanism is also responsible for the rim formation when ablation takes place at higher temperatures. The polymer in the rim is heated only above the glass transition, but not above the ablation threshold, i.e. the polymer only expands at these points.

As expected no rim formation and no photomechanical ablation was observed on the polyimide films since the polymer did not cross a glass transition in the heating phase. Craters similar to the one showed in Figure 6 were observed at the ablation threshold. The behavior of the triazene polymer was, however, surprising. It showed a glass transition at 336 K in differential scanning calorimetry, but no change in surface morphology was observed at a maximum center temperature well above the glass transition temperature at 387 K. At higher temperatures, craters were found corresponding to the ones in Figure 6. The studies of Furutani et al., who could visualize a photothermally induced expansion of UV laser irradiated polymer via nanosecond interferometry (Furutani et al. 1997), as well as the formation of a rim surrounding the ablation craters (Figure 6) suggest, that an expansion took place although it was not detectable. The expanding area might be too large due to the low glass transition temperature, reducing the amount of expansion perpendicular to the surface. Therefore a less pronounced rim was formed, and also no photomechanical ablation took place. The stress built up before the glass transition might simply not be sufficient for ablation.

### Ablation behavior

**Figure 8:**
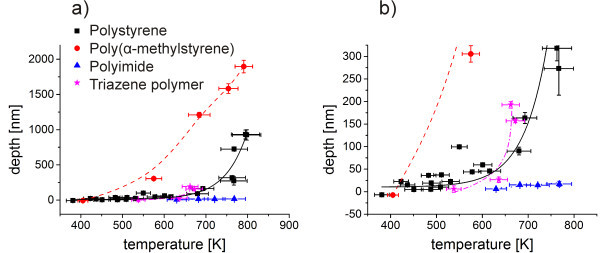
**Depth vs. center temperature for various polymers. (a)** is showing an overview while **(b)** is giving a close up around the ablation threshold. For polystyrene results of films thicknesses between 266 and 1930 nm were included. The temperatures correspond to a delay time of 14 μs. The lines are guides for the eye.

A quantitative comparison of ablation of the different polymers was conducted in Figure 8. The ablation depths and the heights of the deformation peaks prior to ablation were plotted versus the center temperature. Such a plot is independent of film thickness in a relatively wide range as can be seen in the data of polystyrene from films with a thickness of 266 to 1930 nm.

Ablation at the lowest temperatures was found for polystyrene and poly(α-methylstyrene). The photomechanical ablation started at temperatures slightly higher than the ones for surface deformation. For higher temperatures, poly(α-methylstyrene) showed deeper craters than the polystyrene. This agrees well with the lower thermal stability of the polymer. At the threshold temperature, photomechanical ablation prevailed while for higher fluences and temperatures, thermal degradation took over as dominating mechanism.

The polyimide showed the highest threshold temperature and the lowest crater depths at high temperatures. The polymer did not show photomechanical ablation due to the absence of a glass transition between room and degradation temperature. The threshold is consequently shifted to higher temperatures in agreement with the higher thermal stability of the polymer. The triazene polymer did also not show photomechanical ablation. In spite of the low thermal stability, the ablation threshold temperature was at higher temperatures compared to polystyrene or poly(α-methylstyrene). However, at high temperatures the crater depths increased significantly as a consequence of the low thermal stability. Furthermore, two moles of nitrogen form during thermal degradation of the polymer per repeating unit (Lippert et al. 1999; Banks et al. 2008). Therefore, a high pressure can build up in the crater during ablation, leading to an efficient ejection of the material and a pronounced depression of the center temperature. This explains the steep increase in crater depth with temperature as can be seen in Figure 8.

### Threshold temperatures

The maximum center temperatures at the threshold can also be obtained from the shown data. For polystyrene and poly(α-methylstyrene) a temperature slightly above 400 K and 440 K, respectively were determined. Caused by photomechanical ablation, the temperatures can be found 30–40 K above the glass transition temperature. The threshold temperature of the polyimide and the triazene polymer were determined to be 570 K and 510 K, respectively. They were both calculated by extrapolation of the data shown in Figure 8. This agrees well with the start of thermal degradation at low heating rates (Table 1).

For the triazene polymer also the comparison to fast heating rates can be drawn. In the study of Fardel et al. (Fardel et al. 2008), the photochemical ablation behavior of the polymer was analyzed using a 30 ns UV laser pulse. A threshold temperature of about 1300 K was calculated based on a thermal model taking heat conversion and diffusion into account. This is significantly higher than the threshold temperature of 510 K for 15 μs laser pulse obtained via the temperature measurement. Several aspects need to be considered for a meaningful comparison. First of all, in the studies of Fardel et al. UV laser light was used. This might induce a different ablation mechanism. However, in general lower temperatures are expected for photochemical ablation as compared to purely photothermal ablation. Second and most prominent, the pulse duration is reduced by about three orders of magnitude.

In principle, the law of Arrhenius predicts that for shorter time scales, higher temperatures are necessary in order to maintain an equal reaction rate. A higher reaction temperature thus seems legit for the 10 ns laser pulse time scale. However, a quantitative estimation was not possible as a consequence of the unknown activation energy of the reaction, which is required to apply the law of Arrhenius.

The criterium of thermal confinement is more likely to be hit for the short laser pulse, although the absorption depth as well as the film thickness may not be neglected in a direct comparison (Zhigilei et al. 2003; Brygo et al. 2006). On the 10 ns time scale of the short pulse, superheating is possible and necessary for ablation while for the 15 μs laser pulse applied here, no superheating was found prior to ablation.

### Influence of molecular weight

Two films with polystyrene of different molecular weights (M_n_ = 3.900 g/mol resp. 158.000 g/mol) were chosen in order to study the influence of the molecular weight on the ablation behavior. The high molecular weight polymer had a glass transition at 366 K, the low molecular weight polymer at 348 K (DSC, midpoint DIN).

**Figure 9:**
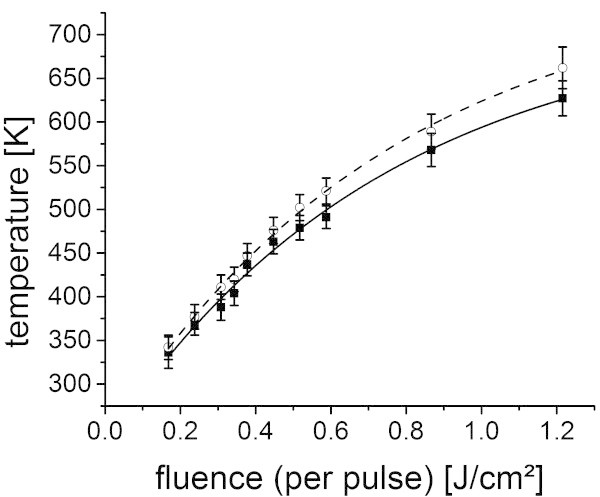
**Center temperature vs. fluence for polystyrene film of high (squares, solid line) and low (circles, dashed line) molecular weight.** Polymer films with a thickness of 2045 nm and absorption per film thickness of 27,300 m^-1^ for the high respectively 1555 nm and 43,700 m^-1^ for the low molecular weight were used.

The films were illuminated at various laser fluences (Figure 9). Films with small differences in absorption and thickness were prepared in order to facilitate a direct comparison. The temperature depends mostly on these two parameters as already discussed in Results and discussion - Laser-polymer interaction as well as in our previous study (Kappes et al. 2011). In agreement with these findings, a similar temperature versus fluence behavior was found for both polymer films, with only slightly higher temperatures for the low molecular weight polymer (Figure 9). In this respect, it is important to notice that this is true for the entire temperature range applied.

### Behavior at the ablation threshold

**Figure 10:**
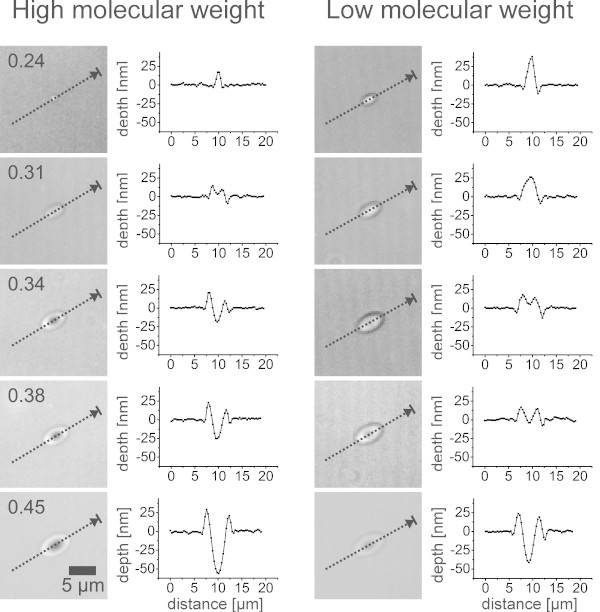
**Surface profiles after laser illumination with different fluences (given in the upper left corner) for high and low molecular weight.**

The surface profiles around the ablation threshold are shown in Figure 10. A deformation of the surface was evident for high and low molecular weights. At low fluence levels a protrusion of 20–40 nm in height appeared on the surface. With increasing fluence the protrusion developed into a crater surrounded by a protruding rim.

The protrusion was more pronounced for the low molecular weight polymer. Furthermore, it increased in diameter while it decreased in height for the next step in fluence (0.31 J/cm^2^). A transition to the typical crater, already presented in Figure 6, was found for both cases at higher fluence. A faster increase in crater depth was apparent for the high molecular weight polymer, while the lateral size including the rim was bigger for the low molecular weight polymer.

These results are caused by the difference in mechanical properties and glass transition temperature and their influence on the photomechanical ablation mechanism. The deformation and dissipation of the stress could take place already at lower temperatures for the low molecular weight polymer. Additionally, the viscosity above the glass transition is lower for the low molecular weight polymer. Therefore, the material had a higher mobility and was able to dissipate higher amounts of the stress, which was built up below the glass transition. As a result a larger surface deformation peak was formed.

The increase in size of the crater and the rim for the low molecular weight polymer film can also be attributed to the lower glass transition temperature. Using finite element simulations it was shown, that in spite of the difference in film thickness, a difference of less than 0.1 μm of the full width half maximum of the temperature profile along the long axis of the laser spot was obtained (simulation with 0.34 J/cm^2^, comparison at delay time of 14 μs). The increase in size was about 1 μm for the low molecular weight, which corresponds to the difference of about 20 K in the temperature profiles, well in agreement to the difference in glass transition temperature between both polymers.

### Ablation behavior

**Figure 11:**
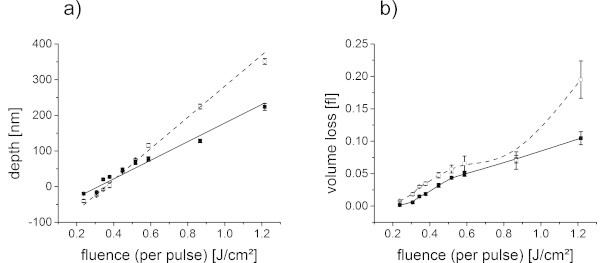
**Ablation behavior in dependence of the fluence.** Depth vs. fluence **(a)** and volumse vs. fluence **(b)** for polystyrene of high (squares, solid line) and low (circles, dashed line) molecular weight. A negative depth corresponds to a protrusion. The lines are guides for the eye.

**Figure 12:**
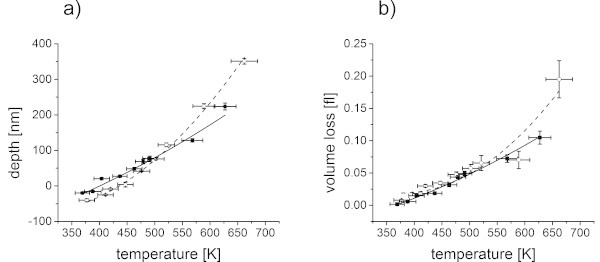
**Ablation behavior in dependence of the temperature.** Depth vs. center temperature **(a)** and volume vs. center temperature **(b)** for polystyrene of high (squares, solid line) and low (circles, dashed line) molecular weight. A negative depth corresponds to a protrusion. The lines are guides for the eye.

If the depth values, resulting from a broad range of fluences, are regarded (Figure 11(a)), an almost linear dependence of the depth on the fluence was found. The slope was steeper for the low molecular weight polymer, i.e. higher protrusion heights at low fluences were evident (compare figure ten) but after a transition zone also deeper craters at high fluences.The volume loss with increasing fluence was analyzed additionally from the 3D profiles, which were acquired with white light profilometer, assuming constant density of the polymer (Figure 11(b)). In this graph, the differences between the low and the high molecular weight polymer were only pronounced at high fluences. No volume loss was found for the lowest fluence, indicating that only a deformation of the surface took place. The volume loss then slowly increased with increasing fluences. While the overall trend of the volume loss was similar, a slightly higher volume loss was detected for the low molecular weight polymer, which can be assigned to the higher temperature at constant fluence (Figure 9).By plotting the depth and the volume loss versus temperature (Figure 12), a similar behavior was found. The depth versus temperature curve of the low molecular weight is again steeper than the one of the high molecular weight polymer, comparable to results in Figure 11(a). At constant temperature, a more pronounced protrusion was evident for the low molecular weight as well as deeper craters at high temperatures. The curves intersect at about 500 K.The volume loss differs insignificantly when plotted versus temperature for both molecular weight polymers (Figure 12(b)). A higher ablation volume was found for the low molecular weight polymer, except for temperatures above 500 K, which is in good agreement to the volume loss versus fluence graph (Figure 11(b)). This underlines the thesis that the higher volume loss for the low molecular weight polymer results from the differences in temperature versus fluence behavior (Figure 9). Hence it was rather caused by small differences in absorption and film thickness than by the difference in molecular weight.In accordance to the discussion about the surface profiles (Figure 10), the differences in behavior can be explained considering the two main differences of the two molecular weight polymers: the mechanical property and the glass transition temperature. Low molecular weight polymers possess a lower viscosity above glass transition. Furthermore, their lower glass transition temperature leads to lower stresses at the transition. They can be deformed easier and at lower temperatures, consequently forming a more pronounced protrusion. At the same time the stress is lower, so the craters formed just above the threshold are not as deep as the ones on the high molecular weight polymer films. If the threshold stress for ablation was however exceeded, the lower viscosity took over as the dominating property, resulting in deeper craters for temperatures above 500 K. The low molecular weight polymer could not only be deformed easier but could also be accelerated more efficiently in a photomechanical ablation process.

At the higher fluence, well above the decomposition temperature from thermogravimetry, thermal decomposition was taking over as the dominating mechanism (Table 1). Both molecular weight polymers were expected to behave similar in a thermal decomposition reaction. Only the number of bonds, which had to be broken in order to produce volatile ablation products, was lower for the low molecular weight polymer than for the high molecular weight derivative and consequently a higher volume loss at high temperatures was obtained.A threshold temperature of about 400 K to 430 K could be derived from the graphs in Figure 12. The differences in the threshold temperature between the two different molecular weight polymers are well within the error of the measurement. However, by considering the explanation of the photomechanical behavior at the threshold (Figure 10), it should be assumed that the low molecular weight polymer has a higher threshold temperature, as it dissipates more energy into deformation before ablation.

## Conclusion

Photothermal laser ablation was studied for several different polymers as well as for different molecular weights in the regime close to thermal confinement by applying a temperature measurement method. In this regime, no indication for superheating prior to ablation could be found. Three polymer properties, the thermal stability, the glass transition behavior and the mechanical properties above glass transition were taken into account to understand the behavior upon laser irradiation. The interpretation of the data was founded on the knowledge of the temperature and the results of standard quasi-equilibrium time scale measurement methods of the individual polymers. We believe that the obtained results contribute significantly to the overall understanding of polymer behavior upon laser irradiation and will help to predict processes in the future, especially those in which a temperature measurement is challenging or even impossible to achieve.
